# Vitamin D Supplementation and Hemoglobin Levels in Hypertensive Patients: A Randomized Controlled Trial

**DOI:** 10.1155/2016/6836402

**Published:** 2016-02-23

**Authors:** Jana B. Ernst, Andreas Tomaschitz, Martin R. Grübler, Martin Gaksch, Katharina Kienreich, Nicolas Verheyen, Winfried März, Stefan Pilz, Armin Zittermann

**Affiliations:** ^1^Clinic for Thoracic and Cardiovascular Surgery, Heart and Diabetes Center NRW, Ruhr University Bochum, Georgstraße 11, 32545 Bad Oeynhausen, Germany; ^2^Department of Cardiology, Medical University of Graz, Auenbruggerplatz 15, 8036 Graz, Austria; ^3^Specialist Clinic for Rehabilitation Bad Aussee, Braungasse 354, 8990 Bad Aussee, Austria; ^4^Department of Cardiology, Charité University, Campus Virchow, Augustenburger Platz 1, 13353 Berlin, Germany; ^5^Department of Internal Medicine, Division of Endocrinology and Metabolism, Medical University of Graz, Auenbruggerplatz 15, 8036 Graz, Austria; ^6^Swiss Cardiovascular Center Bern, Department of Cardiology, Bern University Hospital, 3007 Bern, Switzerland; ^7^Medical Clinic V (Nephrology, Hypertensiology, Endocrinology, Diabetology, Rheumatology) Mannheim Medical Faculty, University of Heidelberg, Theodor-Kutzer-Ufer 1-3, 68167 Mannheim, Germany; ^8^Clinical Institute of Medical and Chemical Laboratory Diagnostics, Medical University of Graz, Auenbruggerplatz 15, 8036 Graz, Austria; ^9^Synlab Academy, Synlab Laboratory Services GmbH, P5, 7, 68161 Mannheim, Germany; ^10^Department of Epidemiology and Biostatistics, EMGO Institute for Health and Care Research, VU University Medical Center, Van der Boechorststraat 7, 1081 BT Amsterdam, Netherlands

## Abstract

Epidemiological evidence suggests that circulating 25-hydroxyvitamin D (25OHD) levels are inversely associated with hemoglobin (Hb) levels and anemia risk. We evaluated whether vitamin D supplementation improves Hb levels and reduces anemia risk in hypertensive patients. Two hundred patients with 25OHD levels <75 nmol/L who attended the Styrian Vitamin D Hypertension Trial were included, of whom 188 completed the trial. Patients randomly received 2800 IU vitamin D3 daily or a matching placebo for eight weeks. Initially, the prevalence of anemic status (Hb levels <12.5 g/dL) and deficient 25OHD levels (<30 nmol/L) was 6.5% and 7.5%, respectively. All anemic patients had 25OHD levels >50 nmol/L. The mean (95% confidence interval) vitamin D effect on Hb levels was 0.04 (−0.14 to 0.22) g/dL (*P* = 0.661). Moreover, vitamin D treatment did not influence anemic status significantly (*P* > 0.999). Likewise, vitamin D had no significant effect on Hb levels in the subgroups of anemic patients or in patients with initial 25OHD levels <30 nmol/L. In conclusion, a daily vitamin D supplement of 2800 IU for eight weeks did not improve Hb levels or anemic status in hypertensive patients. Future trials should focus on anemic patients with deficient 25OHD levels (e.g., <30 nmol/L). This trial is registered with clinicaltrials.gov [NCT02136771].

## 1. Introduction

Anemia is a global health problem [1] and an independent risk factor for increased morbidity and mortality in various groups of patients, especially in patients with chronic diseases and in the elderly [2]. Low levels of 25-hydroxyvitamin D (25OHD) are also highly prevalent among these patients [3].

Recent epidemiological evidence suggests that circulating 25OHD is inversely associated with hemoglobin (Hb) levels [4–15]. The risk of anemia is highest at deficient 25OHD levels (i.e., <30 nmol/L) and lowest at 25OHD levels of 50 to 125 nmol/L [4, 6, 15].

In chronic kidney disease (CKD) patients, some nonrandomized intervention studies could already show that intravenous administration of activated vitamin D (1,25-dihydroxyvitamin D3 = 1,25(OH)_2_D_3_) is associated with an increase in Hb levels within 12 months of treatment and a reduced need for erythropoietin (EPO) [16, 17]. Moreover, intravenous 1,25(OH)_2_D_3_ administration was associated with a decreased weekly EPO dose of 50% [18]. Regarding Hb levels, similar results have been obtained in hemodialysis patients with oral alfacalcidol (1*α*-vitamin D_3_) after 8 weeks [19] and also after 12 and 18 months of treatment [20]. In hemodialysis patients, high-dose oral vitamin D_2_ (50,000 IU monthly) was associated with dose-reductions in erythropoiesis-stimulating agents (ESA), while Hb concentrations remained unchanged [21, 22]. In children with CKD stage 5 and 25OHD levels <75 nmol/L, 12 weeks of vitamin D_2_ treatment in conjunction with 1,25(OH)_2_D_3_ was associated with a significantly reduced dose of ESA required to treat the children [23]. In anemic patients with preserved kidney function, however, one single intramuscular bolus of 600,000 IU vitamin D_3_ did not influence Hb levels [24]. Nevertheless, it is noteworthy that in general populations the effect of high-dose bolus administration of vitamin D on clinical outcomes has been questioned [25].

The purpose of the present study was to determine the effect of a daily vitamin D_3_ supplement on Hb levels in a group of hypertensive patients with preserved kidney function and inadequate 25OHD levels.

## 2. Methods

### 2.1. Study Design

This is a secondary analysis of the Styrian Vitamin D Hypertension Trial of a postspecified endpoint. The study was a randomized, double-blind, placebo-controlled, single-center trial which took place at the outpatient clinic at the Division of Endocrinology and Metabolism, Medical University of Graz, Austria. Major study results have already been published elsewhere [26]. The study was registered at EudraCT (number 2009-018125-70) and clinicaltrials.gov (NCT02136771). All study participants gave written informed consent. The study was approved by the Ethics Committee of the Medical University of Graz, Austria.

### 2.2. Participants

Two hundred study participants (106 men and 94 women) were recruited from the clinics of the Department of Cardiology and the Department of Internal Medicine, Division of Endocrinology and Metabolism, Medical University of Graz, Austria, from June 2011 to August 2014. Eligible study participants were adults aged 18 years or older with a serum 25OHD concentration below 75 nmol/L and arterial hypertension. Pregnant or lactating women were excluded as well as patients with hypercalcemia (serum calcium >2.65 mmol/L), regular vitamin D intake >880 IU per day during the last four weeks before the study, estimated glomerular filtration rate (eGFR) <15 mL/min per 1.73 m^2^, drug intake as part of another clinical study, acute coronary syndrome or cerebrovascular event in the previous two weeks, 24-hour systolic BP >160 mm Hg or <120 mm Hg, 24-hour diastolic BP >100 mm Hg, change in hypertensive treatment (drugs or lifestyle) in the previous four weeks or planned changes in antihypertensive treatment during the study, any disease with an estimated life expectancy of <1 year, any clinically significant acute disease requiring drug treatment, and chemotherapy or radiation therapy.

### 2.3. Intervention

Eligible study participants were randomly allocated to receive 2800 IU (70 *μ*g) cholecalciferol as seven oily drops per day (Oleovit D3, Fresenius Kabi Austria, Graz, Austria) or a matching placebo for eight weeks. The dose of 2,800 IU vitamin D per day was chosen because a rule of thumb suggests that vitamin D supplementation of 1,000 IU increases 25OHD levels by approximately 25 nmol/L [27]. Given that a commonly used normal range of 25OHD is 75 to 150 nmol/L [28] we conclude that a supplementation of 2,800 IU daily may be sufficient to increase the 25OHD level of most study participants to target ranges without causing supraphysiological 25OHD levels. One hundred patients were assigned to the intervention group and 100 patients to the control group. Randomization was performed by web-based software (http://www.randomizer.at/) with a permuted block randomization (block size of 10 and stratification according to sex).

### 2.4. Endpoints

The primary outcome measures were Hb levels and anemia. In accordance with earlier classifications [4, 6, 29], Hb concentrations <12.5 g/dL were considered as anemic, which corresponded to the average threshold value of the World Health Organization gender-based definition (<13.0 g/dL in men and <12.0 g/dL in women).

### 2.5. Biochemical Measurements

Blood sampling was performed in the morning between 7 and 11 am after an overnight fast. All blood samples were either measured at least four hours after blood collection or immediately stored at −20°C until analysis. Measurement of 25OHD was performed by chemiluminescence assay (IDS-iSYS 25-hydroxyvitamin D assay; Immunodiagnostic Systems Ltd., Boldon, UK) on an IDS-iSYS multidiscipline automated analyzer. Lower and upper quantification limits were 17.5 nmol/L and 312.5 nmol/L, respectively. All hematological parameters were measured on a Sysmex® XE-5000 automated hematology analyzer (Sysmex America, Inc., Mundelein, IL, USA). eGFR was calculated using Modification of Diet in Renal Disease [30]. The measurements of other biochemical parameters have been described elsewhere [26]. In accordance with published data [31, 32] we categorized 25OHD levels <30 nmol/L as deficient, 30–49.9 nmol/L as insufficient, and 50 to 74.9 nmol/L as borderline.

### 2.6. Statistics

Categorical variables are reported as a percentage of observations. Normally distributed continuous data are shown as means with standard deviation. We used the Kolmogorov-Smirnov test to check normal data distribution. Normal distribution was a consideration when probability values were >0.05. Variables with a skewed distribution are shown as medians with interquartile range. Change from baseline data is shown as means and 95% confidence interval (CI). We used the McNemar test and Fisher's exact test, respectively, to assess differences in anemic status within and between groups. The unpaired *t*-test or chi-squared test was used for group comparisons at baseline. Skewed variables were normalized by logarithmic transformation before use in parametric statistical analysis. ANCOVA with adjustments for baseline values was used to test for differences in the outcome variables between the vitamin D and the placebo group at the follow-up visit. We considered *P* values < 0.05 (two-sided) as statistically significant. Analyses were performed using SPSS version 21.0 (SPSS, Inc., Chicago, IL, USA).

## 3. Results

Baseline characteristics of the study participants are shown in Table 1. In both groups, mean Hb values were clearly above the anemia threshold of 12.5 g/dL. At baseline, the proportion of anemia in the vitamin D and placebo groups was 9.0% and 4.0%, respectively (*P* = 0.152).

Regarding 25OHD levels, 6.0% of the vitamin D group and 9.0% of the placebo group had deficient levels. The proportion of insufficient 25OHD levels was 27.0% and 36.0%, respectively. In the remaining 67.0% and 55.0%, the vitamin D status was classified as borderline.

Of the 200 participants, 188 terminated the study as planned.  Table 2 shows the treatment results on biochemical parameters.

**Figure 1:**
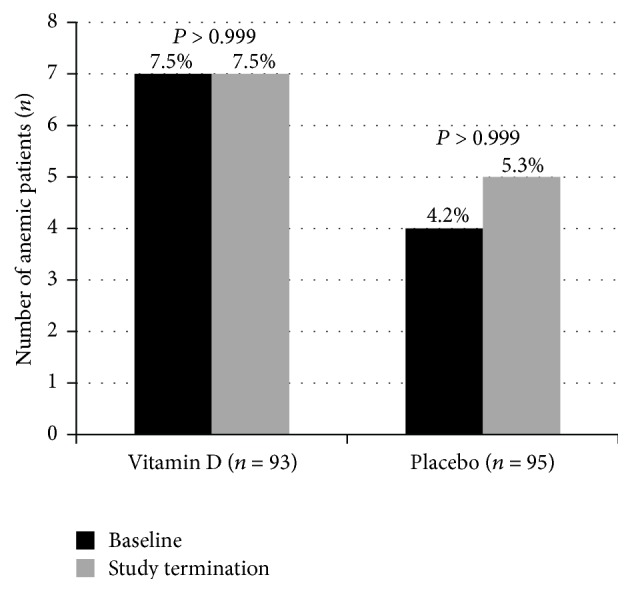
Anemia proportion in participants of the Styrian Vitamin D Hypertension Trial at baseline and study termination (by study group). *P* values indicated within study group results.

Circulating 25OHD increased on average by 35.4 nmo/L (95% CI: 31.2 to 39.6 nmol/L) in the treatment group and 8.1 nmol/L (95% CI: 4.6 to 11.7 nmol/L) in the placebo group (*P* < 0.001). There was no significant vitamin D effect on Hb levels (Table 2). In both study groups, Hb levels remained almost constant. Moreover, vitamin D treatment did not influence anemic status significantly (*P* > 0.999). In detail, the percentage of anemic subjects remained constant in the vitamin D group (*P* > 0.999) and increased only slightly in the placebo group (*P* > 0.999) (Figure 1). Vitamin D treatment had no effect on other hematological parameters (Table 2).

All anemic patients had initial 25OHD levels >50 nmol/L. Vitamin D treatment had no effect on Hb levels and other hematological parameters in anemic patients (see Table S1 in Supplementary Material available online at http://dx.doi.org/10.1155/2016/6836402). Moreover, there was no vitamin D effect on Hb levels and other hematological parameters in the group of subjects with initial 25OHD levels <30 nmol/L (Table S2).

There was, however, a significant vitamin D effect on PTH levels, with suppressed PTH levels in the vitamin D group (Table 2). Vitamin D treatment did not influence serum calcium and phosphate levels.

## 4. Discussion

This study showed that a daily vitamin D supplement of 2800 IU for 8 weeks had no effect on Hb levels or anemia risk in hypertensive patients with 25OHD levels <75 nmol/L.

Our study has several strengths. First, this is a randomized, placebo-controlled trial. In addition, compared with previous interventional studies we were able to include a large number of patients. Next, we included only patients with relatively low circulating 25OHD levels. Moreover, the daily vitamin D dose was sufficient to increase in-study 25OHD levels on average clearly above 75 nmol/L. This value is recommended by many endocrinologists and vitamin D researchers as the lower target level for various clinical outcomes [33, 34]. Our study also has some limitations. First, the prevalence of anemic patients at baseline was low. Second, the study duration of 8 weeks was relatively short, given that the half-life of red blood cells in circulation is 3 months. Third, we have no data on circulating levels of 1,25(OH)_2_D, which is the active, hormonal form of vitamin D.

Generally, evidence is increasing that 1,25(OH)_2_D can stimulate erythropoiesis in red blood cell precursor cells by increasing EPO sensitivity. Furthermore, 1,25(OH)_2_D can upregulate proliferation of hematopoietic progenitor cells [35, 36]. Nevertheless, our findings with plain vitamin D in patients with preserved kidney function do not support the beneficial impact on Hb levels of earlier studies with activated vitamin D in CKD patients [16, 17]. It is, however, noteworthy that in CKD patients the prevalence of anemia is high and circulating 1,25(OH)_2_D levels are low. Moreover, a recent meta-analysis of randomized controlled trials has demonstrated that, after administration of vitamin D or activated vitamin D, the increase in circulating 1,25(OH)_2_D tends to be much higher in CKD patients than in non-CKD patients [37]. In our study, a vitamin D-induced increase in circulating 1,25(OH)_2_D levels may have been blunted by the suppressed PTH levels, and this may have contributed, at least in part, to the null effect on Hb levels.

In the present study, the prevalence of deficient 25OHD levels was low. Moreover, it was a surprising finding that all 13 anemic patients had initial 25OHD levels >50 nmol/L. Generally, epidemiological evidence suggests that circulating 25OHD levels are inversely associated with Hb levels and anemia risk [4, 6]. A recent meta-analysis of retrospective observational studies showed that, compared with individuals with adequate 25OHD levels, low 25OHD levels were associated with an odds ratio for anemia of 2.25 (95% CI: 1.47–3.44) [38]. The cut-off for low 25OHD levels was 50 nmol/L in 5 out of the 7 included studies and 75 nmol/L in the remaining 2 studies. Only individuals without chronic diseases were included in that meta-analysis. However, two large studies in patients with cardiovascular disease [4, 6] indicate that Hb levels are only significantly affected if circulating 25OHD levels are in the deficiency range. Also of note is the fact that observational studies have shown circulating 1,25(OH)_2_D to be a better predictor of anemia than circulating 25OHD [4, 5, 39]. Altogether, data indicate that especially anemic patients with deficient 25OHD levels and low circulating 1,25(OH)_2_D levels may benefit from improved vitamin D status.

## 5. Conclusions

In conclusion, our data demonstrate that a daily vitamin D supplement of 2800 IU for 8 weeks does not increase Hb levels in nonanemic hypertensive patients with 25OHD levels <75 nmol/L. Future studies in this field should focus on anemic individuals with deficient 25OHD levels.

## Supplementary Material

In a group of hypertensive subjects, vitamin D supplementation did not result in changes of hematological parameters, neither if only anemic patients were considered nor if only individuals with initial 25OHD levels <30 nmol/L were included in the analysis.

## Figures and Tables

**Table 1:** Baseline characteristics of the study groups.

Characteristics	Vitamin D group	Placebo group	*P* value
	(*n* = 100)	(*n* = 100)	
Females (%)	46	48	0.777
Age (years)	60.5 ± 10.9	59.7 ± 11.4	0.607
Anemic subjects^1^ (%)	9	4	0.152
BMI (kg/m^2^)	30.4 ± 4.4	30.4 ± 6.2	0.967
Active smoker (%)	19	14	0.341
Previous MI (%)	8	5	0.390
Previous stroke (%)	9	7	0.602
Diabetes mellitus (%)	32	41	0.186
25OHD (nmol/L)	54.5 ± 13.6	51.0 ± 14.2	0.073
PTH (pmol/L)	5.2 (4.2–6.5)	5.5 (4.2–7.0)	0.931
Phosphate (mg/dL)	2.9 ± 0.5	3.0 ± 0.5	0.085
Calcium (mmol/L)	2.37 ± 0.10	2.37 ± 0.11	0.989
Hemoglobin (g/dL)	14.4 ± 1.3	14.4 ± 1.4	0.665
Hematocrit (%)	41.1 ± 3.3	41.5 ± 3.4	0.329
Leukocyte counts (10^9^/L)	5.9 (5.1–7.0)	6.0 (5.0–7.5)	0.817
Erythrocyte counts (10^12^/L)	4.8 ± 0.4	4.9 ± 0.4	0.237
MCV (*µ*m^3^)	86.1 ± 4.4	85.7 ± 4.0	0.457
MCH (pg Hb/red blood cell)	30.1 ± 1.9	29.8 ± 1.6	0.234
MCHC (g/L)	34.9 ± 1.0	34.7 ± 1.1	0.207
Platelets (10^9^/L)	239 ± 64	232 ± 50	0.442
MPV (fl)	10.7 ± 1.0	10.7 ± 0.9	0.572
CRP (mg/L)	2.3 (1.13–3.8)	1.4 (0.9–3.4)	0.044
eGFR (mL/min/1.73 m^2^)	80.0 ± 17.9	77.2 ± 17.9	0.272
Vitamin D supplement (%)	5	9	0.268
ACE-inhibitor (%)	25	38	0.048
AT II blocker (%)	33	31	0.762
Diuretic (%)	42	48	0.394
Beta-blocker (%)	44	49	0.478
Statin (%)	26	32	0.350
Calcium channel blocker (%)	27	25	0.747

^1^Hemoglobin <12.5 g/dL.

25OHD: 25-hydroxyvitamin D; ACE: angiotensin converting enzyme; AT: angiotensin; BMI: body mass index; CAD: coronary artery disease; CRP: C-reactive protein; eGFR: estimated globular filtration rate; MCV: mean corpuscular volume; MCH: mean corpuscular hemoglobin; MCHC: mean corpuscular hemoglobin concentration; MI: myocardial infarction; MPV: mean platelet volume; PTH: parathyroid hormone.

**Table 2:** Results of vitamin D treatment on hematological parameters, calcium and phosphate metabolism, and additional parameters in hypertensive subjects.

Characteristics	Vitamin D group (*n* = 93)			Placebo group (*n* = 95)			Treatment effect	*P* value^2^
	Baseline	Follow-up (8 weeks)	Mean change from baseline^1^	Baseline	Follow-Up (8 weeks)	Mean change from baseline^1^		
Hematological parameters								
Hemoglobin (g/dL)	14.4 ± 1.3	14.3 ± 1.3	−0.1 (−0.18 to 0.05)	14.4 ± 1.4	14.3 ± 1.3	−0.1 (−0.25 to 0.04)	0.04 (−0.14 to 0.22)	0.661
Hematocrit (%)	41.1 ± 3.2	41.1 ± 3.3	0.02 (−0.31 to 0.36)	41.5 ± 3.5	41.3 ± 3.2	−0.2 (−0.59 to 0.19)	0.16 (−0.33 to 0.65)	0.514
Erythrocytes (10^12^/L)	4.8 ± 0.4	4.8 ± 0.4	−0.02 (−0.05 to 0.02)	4.9 ± 0.4	4.8 ± 0.4	−0.04 (−0.09 to 0.00)	0.01 (−0.05 to 0.07)	0.691
MCV (*µ*m^3^)	86.4 ± 4.3	86.7 ± 4.6	0.3 (−0.14 to 0.72)	85.6 ± 4.0	86.0 ± 4.0	0.3 (−0.09 to 0.77)	0.01 (−0.59 to 0.61)	0.971
MCH (pg Hb/RBC)	30.2 ± 1.8	29.9 ± 1.7	−0.04 (−0.16 to 0.08)	29.7 ± 1.6	29.7 ± 1.6	0.05 (−0.07 to 0.18)	−0.08 (−0.25 to 0.09)	0.890
MCHC (g/L)	35.0 ± 1.0	34.8 ± 1.1	−0.2 (−0.3 to 0.00)	34.7 ± 1.1	34.7 ± 1.1	−0.09 (−0.3 to 0.09)	−0.01 (−0.24 to 0.22)	0.938
Leucocytes (10^9^/L)	5.9 (5.1–7.0)	6.0 (5.0–7.2)	0.04 (−0.18 to 0.26)	6.0 (5.0–7.5)	5.8 (5.0–6.9)	−0.1 (−0.31 to 0.12)	−0.13 (−0.17 to 0.42)	0.291
Platelets (10^9^/L)	237 ± 62	240 ± 64	2.9 (−1.6 to 7.4)	231 ± 50	232 ± 53	1.7 (−3.8 to 7.1)	1.6 (−5.4 to 8.6)	0.651
Mean platelet volume (fl)	10.7 ± 1.0	10.7 ± 0.9	0.01 (−0.08 to 0.09)	10.8 ± 0.9	10.8 ± 0.9	−0.02 (−0.1 to 0.08)	0.01 (−0.12 to 0.13)	0.887
Calcium and phosphate metabolism								
25-Hydroxyvitamin D (nmol/L)	54.9 ± 13.6	90.3 ± 18.3	35.4 (31.2 to 39.6)	50.8 ± 14.2	59.0 ± 22.1	8.1 (4.6 to 11.7)	28.7 (23.5 to 34.2)	<0.001
Parathyroid hormone (pmol/L)	5.2 (4.3–6.5)	4.8 (4.0–5.8)	−0.4 (−0.69 to −0.17)	5.4 (4.1–6.8)	5.3 (4.1–7.0)	0.18 (−0.13 to 0.49)	−0.60 (−0.99 to −0.22)	0.003
Phosphate (mg/mL)	2.9 ± 0.5	3.0 ± 0.5	0.1 (0.02 to 0.22)	3.0 ± 0.5	3.1 ± 0.5	0.09 (−0.02 to 0.19)	−0.01 (−0.14 to 0.11)	0.823
Calcium (mmol/L)	2.37 ± 0.1	2.37 ± 0.1	0.00 (−0.02 to 0.02)	2.37 ± 0.1	2.35 ± 0.1	−0.01 (−0.03 to 0.01)	−0.01 (−0.03 to 0.01)	0.259
Additional parameters								
C-reactive protein (mg/L)	2.1 (0.9–3.8)	2.2 (1.0–4.2)	0.3 (−0.6 to 1.2)	1.4 (0.8–3.0)	1.4 (0.6–4.1)	0.2 (−0.2 to 0.6)	0.15 (−0.69 to 1.2)	0.598
eGFR (mL/min/1.73^2^)	80.5 ± 18.1	77.9 ± 18.8	−2.7 (−5.0 to −0.4)	77.5 ± 17.6	76.7 ± 16.9	−0.9 (−3.0 to 1.3)	−1.3 (−4.3 to 1.7)	0.398

^1^Change from baseline data is shown as means and 95% confidence interval.

^2^ANCOVA with adjustments for baseline values was used to test for differences in the outcome variables between the vitamin D and the placebo group.

MCV: mean corpuscular volume; MCH: mean corpuscular hemoglobin; Hb: hemoglobin; RBC: red blood cell; MCHC: mean corpuscular hemoglobin concentration; eGFR: estimated glomerular filtration rate.
