# Supplementary material for: Vitamin D Supplementation and Hemoglobin Levels in Hypertensive Patients: A Randomized Controlled Trial
Source: Int J Endocrinol. 2016 Feb 23;2016:6836402. doi: 10.1155/2016/6836402 (PMC4781958; doi:10.1155/2016/6836402)
Supplement: Supplementary file 1 — In a group of hypertensive subjects, vitamin D supplementation did not result in changes of hematological parameters, neither if only anemic patients were considered nor if only individuals with initial 25OHD levels <30 nmol/L were included in the analysis. [file 6836402.f1.pdf]

Supplemental Material

Table S1: Effect of vitamin D treatment on hematological parameters in anemic hypertensive subjects

Characteristics	Vitamin D group (n=7)			Placebo group (n=4)			Treatment Effect	P-value
	Baseline	Follow-Up (8 weeks)	Mean Change from Baseline	Baseline	Follow-Up (8 weeks)	Mean Change from Baseline		
Hematological Parameters								
Hemoglobin (g/dL)	11.8±0.7	11.8±0.9	-0.01 (-0.64 to 0.61)	10.8±1.3	10.9±1.0	0.15 (-1.74 to 2.04)	0.29 (-1.10 to 1.67)	0.643
Hematokrit (%)	34.5±3.0	34.3±3.3	-0.10 (-1.59 to 1.34)	31.6±3.2	32.4±2.0	0.83 (-3.46 to 5.11)	-0.15 (-3.35 to 3.06)	0.919
Erythrocytes (10^{12}/L)	3.89±0.6	3.91±0.7	0.01 (-0.19 to 0.21)	4.02±0.3	4.04±0.3	0.23 (-0.46 to 0.51)	0.00 (-0.38 to 0.38)	0.985
MCV (μm^3)	89.6±9.0	89.7±11.9	0.13 (-2.85 to 3.10)	78.5±5.0	80.3±3.9	1.75 (-1.75 to 5.20)	-4.14 (-8.50 to 0.23)	0.060
MCH (pg Hb/RBC)	28.8±1.2	28.5±1.3	-0.30 (-0.77 to 0.17)	26.8±2.4	27.0±2.1	0.28 (-1.35 to 1.90)	-0.28 (-1.93 to 1.37)	0.677
MCHC (g/L)	34.3±1.5	34.4±1.2	0.06 (-0.54 to 0.65)	34.0±1.5	33.6±1.3	-0.42 (-3.33 to 2.50)	0.62 (-0.81 to 2.06)	0.345

Table S2: Effect of vitamin D treatment on hematological parameters in hypertensive subjects with initial 25-hydroxyvitamin D levels <30 nmol/l

Characteristics	Vitamin D group (n=6)			Placebo group (n=8)			Treatment Effect	P-value
	Baseline	Follow-Up (8 weeks)	Mean Change from Baseline	Baseline	Follow-Up (8 weeks)	Mean Change from Baseline		
Hematological Parameters								
Hemoglobin (g/dL)	14.7±1.3	14.4±1.1	-0.28 (-0.82 to 0.26)	15.0±1.2	14.5±1.3	-0.46 (-0.80 to 0.11)	0.16 (-0.39 to 0.71)	0.543
Hematokrit (%)	41.7±3.4	40.6±2.7	-1.15 (-2.55 to 0.25)	43.3±2.9	41.7±3.1	-1.52 (-2.67 to 0.37)	0.15 (-1.49 to 1.80)	0.841
Erythrocytes (10^{12}/L)	4.76±0.3	4.63±0.2	-0.12 (-0.23 to 0.02)	5.04±0.5	4.84±0.4	-0.19 (-0.30 to 0.07)	-0.02 (-012 to 0.16)	0.750
MCV (μm^3)	87.6±4.4	87.4±4.2	-0.11 (-1.42 to 1.19)	86.0±4.8	86.1±4.8	0.11 (-2.02 to 2.25)	-0.05 (-2.62 to 2.53)	0.969
MCH (pg Hb/RBC)	30.9±2.2	31.0±2.2	0.18 (-0.56 to 0.92)	29.2±1.7	29.2±1.9	0.08 (-0.26 to 0.42)	0.06 (-0.70 to 0.81)	0.869
MCHC (g/L)	35.1±0.9	35.4±0.6	0.28 (-0.31 to 0.88)	34.4±1.2	34.66±1.1	0.14 (-0.77 to 1.04)	0.48 (-0.49 to 1.46)	0.299
